# Impact of the Gut Microbiota on Intestinal Immunity Mediated by Tryptophan Metabolism

**DOI:** 10.3389/fcimb.2018.00013

**Published:** 2018-02-06

**Authors:** Jing Gao, Kang Xu, Hongnan Liu, Gang Liu, Miaomiao Bai, Can Peng, Tiejun Li, Yulong Yin

**Affiliations:** ^1^National Engineering Laboratory for Pollution Control and Waste Utilization in Livestock and Poultry Production, Institute of Subtropical Agriculture, The Chinese Academy of Sciences, Changsha, China; ^2^Key Laboratory of Agro-Ecology, Institute of Subtropical Agriculture, The Chinese Academy of Sciences, Changsha, China; ^3^University of Chinese Academy of Sciences, Beijing, China; ^4^College of Life Science, Hunan Normal University, Changsha, Hunan, China

**Keywords:** gut microbiota, Trp metabolism, intestinal immunity, intestinal inflammation, aryl hydrocarbon receptor

## Abstract

The gut microbiota influences the health of the host, especially with regard to gut immune homeostasis and the intestinal immune response. In addition to serving as a nutrient enhancer, L-tryptophan (Trp) plays crucial roles in the balance between intestinal immune tolerance and gut microbiota maintenance. Recent discoveries have underscored that changes in the microbiota modulate the host immune system by modulating Trp metabolism. Moreover, Trp, endogenous Trp metabolites (kynurenines, serotonin, and melatonin), and bacterial Trp metabolites (indole, indolic acid, skatole, and tryptamine) have profound effects on gut microbial composition, microbial metabolism, the host's immune system, the host-microbiome interface, and host immune system–intestinal microbiota interactions. The aryl hydrocarbon receptor (AhR) mediates the regulation of intestinal immunity by Trp metabolites (as ligands of AhR), which is beneficial for immune homeostasis. Among Trp metabolites, AhR ligands consist of endogenous metabolites, including kynurenine, kynurenic acid, xanthurenic acid, and cinnabarinic acid, and bacterial metabolites, including indole, indole propionic acid, indole acetic acid, skatole, and tryptamine. Additional factors, such as aging, stress, probiotics, and diseases (spondyloarthritis, irritable bowel syndrome, inflammatory bowel disease, colorectal cancer), which are associated with variability in Trp metabolism, can influence Trp–microbiome–immune system interactions in the gut and also play roles in regulating gut immunity. This review clarifies how the gut microbiota regulates Trp metabolism and identifies the underlying molecular mechanisms of these interactions. Increased mechanistic insight into how the microbiota modulates the intestinal immune system through Trp metabolism may allow for the identification of innovative microbiota-based diagnostics, as well as appropriate nutritional supplementation of Trp to prevent or alleviate intestinal inflammation. Moreover, this review provides new insight regarding the influence of the gut microbiota on Trp metabolism. Additional comprehensive analyses of targeted Trp metabolites (including endogenous and bacterial metabolites) are essential for experimental preciseness, as the influence of the gut microbiota cannot be neglected, and may explain contradictory results in the literature.

## Introduction

The gut microbiota and microbial metabolites are important for maintaining healthy bowels. Although the high complexity of the gut microbial composition and metabolites has presented significant research challenges, direct and indirect evidence of correlations between the gut microbiota, microbial metabolites, and intestinal immune function have been demonstrated, largely through the application of modern molecular biology techniques.

The gut microbiota can influence the scope and quality of the immune system response; in turn, the immune system participates in regulating the localization and composition of the gut microbiota. Recent studies have emphasized the profound effects of diet and nutrients on the localization and composition of the gut microbiota as well as on the connection between the gut microbiota and immunological pathways (Thorburn et al., 2014). As an essential nutrient in mammals, L-tryptophan (Trp), and its endogenous metabolites are involved in gut immune homeostasis and in several immune diseases.

Manipulating the gut microbial composition can modulate plasma concentrations of Trp and Trp metabolites (Clarke et al., 2014). In this review, we discuss studies that increase our understanding of how Trp metabolism interacts with the gut microbiota, how Trp functions in host–microbiota interactions and how Trp influences gut immune homeostasis. We provide a brief outline of studies that support an influence of dietary Trp on intestinal inflammation and other peripheral inflammation, outline the connection between Trp metabolism and the gut microbiota, and discuss which Trp metabolites intersect with the immune system. Understanding these interactions may provide novel targets for the treatment of various intestinal disorders that are associated with the microbiota and Trp metabolism.

## Gut microbiota and host immunity

The microbiome, consisting of microbes and their collective genomes, modulates the host metabolic phenotype and influences the host immune system (Gordon, 2012). Interactions between the gut microbiota and the host immune system begin at birth: the microbiota influences the development of the immune system; and the immune in turn system shapes the composition of the gut microbiota (Nicholson and Wilson, 2003). Later in life, the gut microbiota also influences immune cell recruitment and initiates inflammation. Crosstalk between the gut microbiota and enterocytes shapes the gut environment and profoundly influences intestinal immune homeostasis (Hold, 2016), which lasts a lifetime. Alterations in the gut microbiota, coupled to increased gut permeability (leaky gut), are widely recognized as relevant to the pathogenesis of several diseases, including autoimmune and neurodegenerative disorders (Anderson et al., 2016).

On the one hand, the host immune system is affected by the intestinal microbiome. The connection between microbes and the host immune system is mediated by a series of molecules (Anders et al., 2013) and signaling processes, which can impact the gut, liver, brain, and other organs. Complex host–microbe metabolic axes offer a lasting influence on metabolic reactions, the host immune system, and long-term health outcomes (Blumberg and Powrie, 2012; Cerf-Bensussan and Eberl, 2012; Hooper et al., 2012).

On the other hand, the intestinal immune system plays a crucial role in exposing bacteria to host tissues, alleviating the potential for pathologic outcomes and determining the stratification of intestinal bacteria on the luminal side of the epithelial barrier (Blumberg and Powrie, 2012). Moreover, the immune system controls the composition of the gut microbiota, and at the same time, resident microbes provide signals that foster normal immune system development and regulate ensuing immune responses. Disruption of these dynamic interactions may have far-reaching effects on host health (Hamard et al., 2007).

## Endogenous and bacterial Trp metabolism

### Endogenous Trp metabolism

Trp absorption in the intestine is primarily mediated by B^0^AT1 (SLC6A19). In addition to serving as a substrate for protein synthesis, Trp is primarily metabolized through two metabolic pathways: the kynurenine pathway (KP) and the serotonin pathway. Approximately 95% of the Trp ingested is degraded to kynurenine, kynurenic acid (KA), quinolinic acid, picolinic acid, and nicotinamide adenine dinucleotide (NAD) through KP, which is regulated by two rate-limited enzymes: tryptophan 2,3-dioxygenase (TDO) in the liver and indoleamine 2,3-dioxygenase (IDO) in extrahepatic tissues (Peters, 1991). Specifically, Trp is degraded to kynurenine, which is then largely metabolized to 3-hydroxykynurenine by kynurenine hydroxylase and marginally metabolized to anthranilic acid (AA) by kynureninase and KA by kynurenine aminotransferase. Furthermore, 3-hydroxykynurenine is mainly degraded to 3-hydroxyanthranilic acid by kynureninase and marginally degraded to xanthurenic acid (XA) by kynurenine aminotransferase. Through multi-stage enzymatic reactions, 3-hydroxyanthranilic acid is converted to quinolinic acid, pyridine carboxylic acids (such as picolinic acid, acetyl CoA), nicotinic acid, NAD^+^, and other active molecules (Badawy, 2015).

Approximately 1–2% of ingested Trp is converted to serotonin (5-HT) and melatonin via the serotonin pathway. 5-HT is synthesized from Trp through two-stage enzymatic reactions involving Trp hydroxylase (TPH) and aromatic amino acid decarboxylase. In animals, serotonin is primarily found in the gastrointestinal tract (GI tract), blood platelets, and the central nervous system. Approximately 90% of total serotonin in humans is located in enterochromaffin cells in the GI tract, where it can promote intestinal peristalsis (Bai et al., 2017). Melatonin (N-acetyl-5-methoxytryptamine) is derived from 5-HT via two-step enzymatic conversion reactions (acetylation and methylation) mainly in the pineal gland but also in other tissues such as the retina, GI tract, skin, and leukocytes (Radogna et al., 2010). Although endogenous Trp metabolites play important roles in regulating gut immune homeostasis in mammals, the potential contribution to intestinal immune function by Trp metabolites from resident microbiota should not be ignored.

### Bacterial Trp metabolism

**Figure 1:**
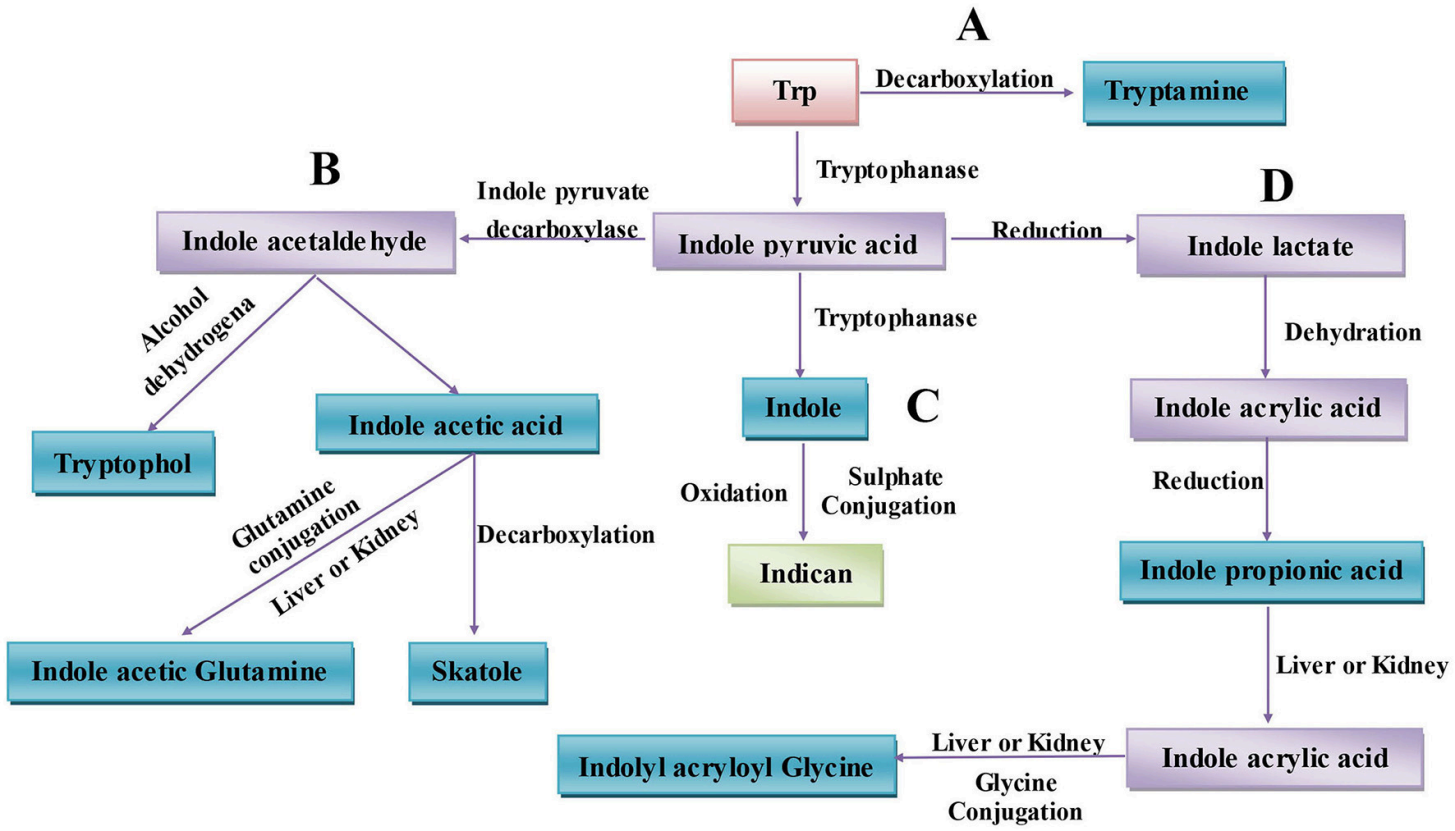
Microbiota-associated tryptophan metabolism in the gut. **(A)** Trp is decarboxylated to tryptamine by the common gut Firmicutes *Clostridium sporogenes* and *Ruminococcus gnavus*. **(B)** Derivatization of indole pyruvic acid from Trp is catalyzed by tryptophanase, and then indole pyruvic acid is decarboxylated to indole acetaldehyde, which is the precursor of tryptophol and indole acetic acid. Indole acetic acid can be converted to skatole by *Lactobacillus, Clostridium, Bacteroides*, and others. **(C)** Indole pyruvic acid can be catalyzed to indole by tryptophanase; after absorption, indole is oxidized to indoxyl, conjugated with sulfate and excreted as urinary indican. **(D)** Indole pyruvic acid can also be converted to indole lactate, to indole acrylic acid, and to indole propionic acid by intestinal microorganisms. Indole propionic acid can be further converted to indole acrylic acid in the liver or kidney and combined with glycine to produce indolyl acryloyl glycine.

The gut microbiota can directly utilize Trp, which partially limits Trp availability for the host. Approximately 4–6% of Trp is metabolized to indole, indican, tryptamine, and skatole as well as indole acid derivatives by the gut microbiota (Figure 1) (Yokoyama and Carlson, 1979). Intestinal microorganisms convert Trp to tryptamine (Figure 1A) and indole pyruvic acid and indole pyruvic acid to indole (Figure 1C), indole acetaldehyde (Figure 1B), and indole lactate (Figure 1D). Indole acetaldehyde can be converted to indole acetic acid and tryptophol, and the former can then be converted to skatole (Figure 1B). Indole lactate may be converted to indole acrylic acid and subsequently to indole propionic acid (Figure 1D) (Smith and Macfarlane, 1997). Although the conversion of these bacterial Trp metabolites are easily defined at the molecular level, it is in practice complicated to determine which type of metabolites are produced. Because different microbes possess different catalytic enzymes, mutual cooperation among more than two bacteria is needed to generate one metabolite from Trp. Unlike the relatively simple background of animal endogenous Trp metabolism, the intestinal environment is relatively complex with regard to bacterial Trp metabolism. Many strains that possess catalytic enzymes for Trp metabolism remain unknown, and research on the coordination of different species of bacteria in generating Trp metabolites is needed. Indeed, before designing strategies to manipulate Trp bacterial metabolism, differences in the gut microbiota and the intestinal environment among individuals should be evaluated.

### Interplay between endogenous and bacterial Trp metabolism

The microbiota can directly and indirectly modulate host endogenous Trp metabolism, and variations in Trp metabolism can negatively influence microbial proliferation and microbiota diversity. Reports have shown that the bacterial community can influence Trp metabolism and the serotonergic system. The balance between bacterial Trp metabolism and Trp synthesis determines local GI and circulating Trp availability for the host.

Circulating total Trp levels are increased in germ-free (GF) mice that lack gut microbiota (Wikoff et al., 2009; Clarke et al., 2012, 2013; El Aidy et al., 2012; Mardinoglu et al., 2015); KP metabolism and circulating 5-HT concentrations are also decreased (Wikoff et al., 2009; Clarke et al., 2013). This may be attributable to gut microbial metabolites such as short-chain fatty acids or Trp-derived indole metabolites, which may promote colonic 5-HT production to modulate circulating Trp levels (Reigstad et al., 2015; Yano et al., 2015).

In addition to being a rate-limited enzyme in KP, IDO1 also plays an essential role in maintaining microbial diversity (Le Floc'h et al., 2011). Host Trp depletion resulting from IDO1 activation can reduce microbial proliferation, and IDO1-induced depletion of Trp caused by host immune activation may lead to microbial amino acid deprivation and immune tolerance. For example, increased production of bacterial Trp metabolites was detected in IDO1-knockout mice (Zelante et al., 2013). In particular, dietary Trp insufficiency alters gut microbial composition and impairs intestinal immunity in mice (Hashimoto et al., 2012). Host Trp modulation in the microenvironment is presumed to involve arrest of microbial proliferation, providing a significant benefit for the host (Le Floc'h et al., 2011). Altering either the gut microbial composition during the host's lifespan or the trajectory of microbial colonization of the GI tract early in life can modulate Trp metabolism.

## Host immune-microbiome interactions associated with Trp metabolism

### Trp

Trp has proven to exert anti-inflammatory effects in mammals, and Trp and its regulatory pathway act as important regulators of inflammatory responses (Marsland, 2016). Mice fed a low-Trp diet are more susceptible to chemically induced inflammation (Hashimoto et al., 2012). Conversely, mice or piglets fed a sufficient-Trp diet had reduced inflammation and decreased severity of dextran sodium sulfate (DSS)-induced colitis (Kim et al., 2010; Zelante et al., 2013; Etienne-Mesmin et al., 2017). Moreover, mice fed a Trp-depleted diet had more severe central nervous system inflammation compared with mice fed a Trp-rich diet, and this manifestation was ameliorated after feeding a diet supplemented with Trp. These effects of dietary Trp on mammalian immunity can be attributed to the production of Trp metabolites.

### Aryl hydrocarbon receptor (AhR) signaling

AhR, a cytosolic ligand-activated transcription factor that mediates xenobiotic metabolism, is a critical regulator of immunity and inflammation, involving fine-tuning of adaptive immunity and mucosal barrier function, maintenance of intestinal homeostasis, and carcinogenesis (Hubbard et al., 2015b; Korecka et al., 2016). The function of AhR signaling in the GI tract has been reported. In DSS-inducible intestinal injury models, AhR-null mice exhibit severe symptoms and mortality (Arsenescu et al., 2011; Benson and Shepherd, 2011), and in another study of intestinal disease models, 2,3,7,8-tetrachlorodibenzo-*p*-dioxin (TCDD)-induced AhR activation decreased lethality and symptom severity (Takamura et al., 2010). *Ahr*^−/−^ mice are more susceptible to intestinal challenge, indicating the critical role of AhR in maintaining gut immune and barrier functions (Sutter et al., 2011). Similarly, in the absence of AhR, mice show high disease susceptibility when infected by *Listeria monocytogenes* and *Citrobacter rodentium* (Shi et al., 2007; Qiu et al., 2012). The promotional effect of AhR on immune homeostasis is usually ascribed to two mechanisms. First, an antimicrobial role for AhR due to AhR-dependent IL-22 transcription [AhR mediates activation of innate lymphoid cell 3 (ILC3) to produce IL-22 in the gut] has been reported (Lee et al., 2012; Qiu et al., 2012); in the gut, IL-22 can regulate the release of antimicrobial peptides and affect the homeostatic balance between immunity and the microbiota by regulating microbial composition (Zelante et al., 2013; Zenewicz et al., 2013; Behnsen et al., 2014). Second, there is evidence for an anti-inflammatory role for AhR mediated by its effects on regulating the development of intraepithelial lymphocytes and innate lymphoid cells (Zelante et al., 2014; Hubbard et al., 2015b). These cells play important roles in defending against infiltrating pathogenic microbes and facilitating gut homeostasis (Hubbard et al., 2015b).

**Figure 2:**
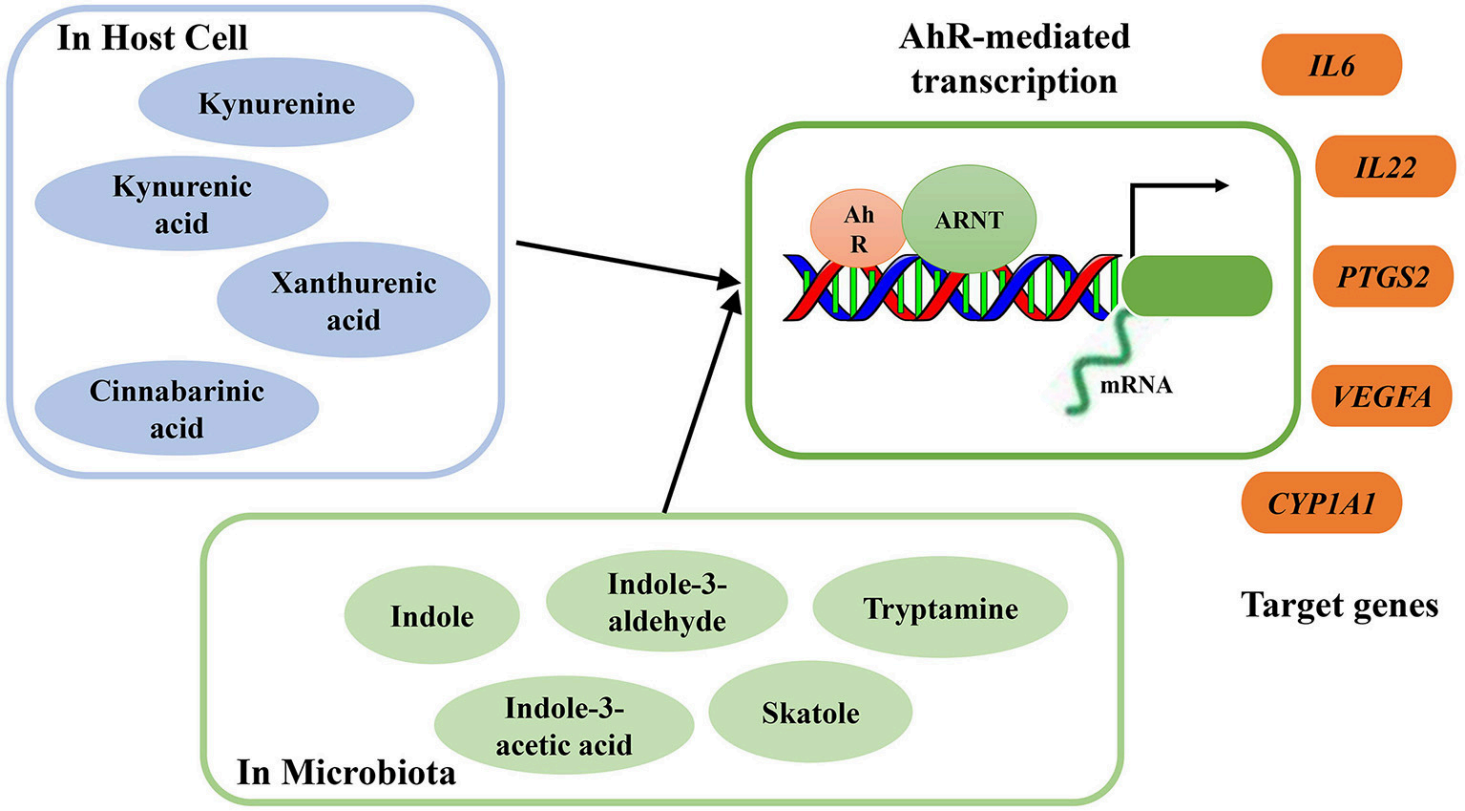
Summary of endogenous and bacterial tryptophan metabolites as AhR ligands. AhR nuclear translocator protein (ARNT), AhR activation and transcription of target genes interleukin-6 (*IL-6*), interleukin-22 (*IL-22*), prostaglandin G/H synthase 2 (*PTGS2*), vascular endothelial growth factor A (*VEGFA*), cytochrome P450 1A1 (*CYP1A1*).

As ligands of AhR, several microbial metabolites are vital to host immunity, especially in protecting the mucosa from inflammation (Rooks and Garrett, 2016). Excessive degradation of AhR ligands induces harmful effects on intestinal immunity, and these effects can be counterbalanced by increased supplementation of dietary AhR ligands (Schiering et al., 2017). Through exposure to AhR ligands, AhR can directly target and activate certain genes. During inflammation, targeted genes, including interleukin-6 (*IL-6*), interleukin-22 (*IL-22*), prostaglandin G/H synthase 2 (*PTGS2*), vascular endothelial growth factor A (*VEGFA*), and cytochrome P450 1A1 (*CYP1A1*), can be regulated by AhR activation (Figure 2). Moreover, consumption of AhR ligands can reverse AhR-mediated regulation of intestinal homeostasis (Hubbard et al., 2015b).

A Trp-rich diet can increase AhR mRNA expression and activate AhR and subsequently increase colonic *IL-22* mRNA expression. During acute colitis, Trp supplementation protects the epithelial layer and prevents the intestinal inflammation mediated by AhR signaling (Hashimoto et al., 2012). In a DSS-inducible intestinal injury murine model, dietary Trp alleviated colitis symptoms and severity through the activation of AhR (Islam et al., 2017). However, the effects of dietary Trp are mediated by its metabolites, which act as AhR ligands, and not by Trp itself (Opitz et al., 2011). Some endogenous and bacterial Trp metabolites have been proven to act as AhR ligands, and their binding activates AhR to regulate intestinal immunity (Zelante et al., 2013; Cheng et al., 2015). Endogenous Trp metabolites such as kynurenine, KA, XA, and cinnabarinic acid (CA) can function as direct AhR ligands, with the capacity to stimulate AhR-dependent gene expression (Romani et al., 2014) (Figure 2). After activation, AhR mediates transcription of IL-22 in human and murine CD4^+^ T-cells (Lowe et al., 2014). Several bacterial Trp metabolites, including indole, indole propionic acid, indole acetic acid, skatole, and tryptamine, have also been proven to be AhR ligands (Bittinger et al., 2003; Chung and Gadupudi, 2011) (Figure 2).

AhR and IDO1 play crucial roles in connecting microbial Trp catabolism and host endogenous Trp metabolites with regulatory T-cell function, especially in AhR-dependent T-cell immune homeostasis at the mucosa. When induced by proinflammatory cytokines, IDO1 is activated, and kynurenines are produced. Acting as AhR ligands, kynurenines regulate immune homeostasis and induce the generation of regulatory T-cells, which protect mice from hyper-inflammatory responses (Bessede et al., 2014). The coevolutionary commensalism between host and microbes may be relevant to the AhR ligands catabolized from Trp (Hubbard et al., 2015b). The absence of IDO1 induces upregulation of commensal-driven AhR/IL-22 activity, but AhR stimulation may in turn affect IDO1 (Bessede et al., 2014). The positive feed-forward loop between IDO1 and AhR is necessary for driving commensal fungi to coevolve with the mammalian immune system and microbiota, which is beneficial for host survival and fungal commensalism under strong inflammatory conditions and prevents dysregulated immunity (Romani et al., 2014).

The generation of AhR ligands and AhR activation is influenced by several factors such as diet, gut microbial composition, and endogenous enzymatic activity (Hubbard et al., 2015b). Through AhR, microbial Trp metabolites may provide important cues to the host for resisting colonization and defense against mucosal inflammation. *Lactobacilli* spp can metabolize Trp to produce AhR ligands, such as indole-3-aldehyde (IAld), which can active innate lymphoid cells (ILCs) (Zelante et al., 2013), after which mucosal resistance against the potential pathogen *Candida albicans* is increased. At the same time, ILC-induced IL-22 production can regulate release of antimicrobial peptides in the gut epithelium and enhance AMP expression, which reduces pathogen infectivity via sequestration of metal ions (Zelante et al., 2014; Rooks and Garrett, 2016).

Previous studies have highlighted the bidirectional interaction between AhR and the microbiome, and the microbiome-AhR axis can influence host metabolism (Korecka et al., 2016). Microbial metabolites such as short-chain fatty acids (SCFAs) and several microbial Trp metabolites can activate AhR and AhR target genes in the intestine or liver. In turn, AhR signaling can influence microbial composition in the small intestine. In addition, AhR acts an important regulator of host-microbiota communication, which can influence host metabolism and modulate the immune system.

### Host Trp metabolism

#### IDO1

As the rate-limiting enzyme of KP, IDO1 also plays an important role in regulating the adaptive immunity of vertebrate hosts (Zelante et al., 2013). When attempting to counterbalance tissue damage, high expression of IDO1 by intestinal mononuclear cells can mediate anti-inflammatory and immunosuppressive effects of IDO1 on the intestinal mucosa (Wolf et al., 2004) by regulating host immunomodulatory activity via kynurenine production, mucosal amino acid nutrition, mucosal immune reactivity, and gut microbial community metabolism (Dai and Zhu, 2010). Moreover, through the influence of IDO1 on T-cells, KP may also serve as the basis of the sensitive balance between pro-inflammatory (excitotoxic quinolinic acid) and anti-inflammatory (neuroprotective KA) states in the GI tract (Kaszaki et al., 2008). This balance can affect intestinal motor or sensory function through a profound influence on the excitability of enteric neurons. In turn, inflammatory mediators tightly regulate KP (Campbell et al., 2014). Specifically, inflammatory cytokines and interferon (IFN)-γ induce expression of IDO1 in the GI tract and other tissues. In addition, the severity of the inflammation induced is linked to the translocation of β-catenin from the cell membrane to the cytoplasm/nucleus (Cooper et al., 2000). Impairment of IDO1 activity, which is detected in inflamed or neoplastic intestinal epithelial cells, can reduce nuclear β-catenin and cell proliferation (Thaker et al., 2013).

In addition, IDO1 is an important activating enzyme in host-microbiota symbiotic relationships via regulation of Trp metabolism (Niño-Castro et al., 2014; Romani et al., 2014). The gut microbiota may influence host Trp degradation and circulating Trp concentrations through KP (O'mahony et al., 2015). In GF animals, KP metabolism (kynurenine:Trp ratio) is decreased due to the microbiota deficiency (Clarke et al., 2013), and colonization by a normal microbiota in GF animals increases KP metabolism (kynurenine:Trp ratio) and reduces plasma Trp. In microbiota-deficient animals, KP cannot be detected in the central nervous system (CNS), but mice infected with Toxoplasma gondii have increased levels of kynurenine, KA, 3-hydroxykynurenine and quinolinic acid in brain tissue (Notarangelo et al., 2014). In contrast, colonization by *Bifidobacteria infantis* in rodents increases Trp levels, circulating KA and the KA:kynurenine ratio and decreases the kynurenine:Trp ratio, suggesting reduced activity of IDO and Trp metabolism through KP, with no effect on kynurenine concentrations (Desbonnet et al., 2008). This increase in KA and decrease in IDO activity appear to conflicting, and other factors need to be taken into account when considering the influence of *B. infantis* colonization on host Trp metabolism. Colonization by *Lactobacillus johnsonii* in rats also decreases ileum IDO mRNA levels and serum kynurenine concentrations, consistent with the effect of *L. johnsonii* culture cell-free supernatant in reducing IDO1 activity in HT-29 intestinal epithelial cells (47% reduction) (Freewan et al., 2013; Valladares et al., 2013). As an explanation, *L. johnsonii* feeding was proven to alter the distribution of ileum and colon IDO1 in rats, and increased ileum lumen H_2_O_2_ produced by *L. johnsonii* was found to be a strong inhibitor of IDO1 activity. The signaling molecule H_2_O_2_ possibly mediated host-microbiota symbiotic interactions. Moreover, the impact of altered IDO1 activity on the degradation of 5-HT is also important, as lower IDO activity leads to both decreased kynurenine and increased 5-HT concentrations.

There is also a correlation between IDO and inducible nitric oxide synthase (iNOS). On the one hand, the NO produced by iNOS inhibits IDO activity by direct interaction or by stimulating IDO degradation. On the other hand, 3-hydroxyanthranilic acid, a kynurenine metabolite, inhibits the expression and catalytic activity of iNOS. Other kynurenine metabolites, quinolinic, and picolinic acids, can also enhance IFN-γ-dependent iNOS expression (Xu et al., 2017). iNOS is involved in the immune response after gut microbiota exposure and helps to limit inflammation. During the inflammatory response, leukocyte recruitment, and adhesion are regulated by iNOS. iNOS-derived NO is maintained at high levels, which is considered a host-protective effect. Similarly, commensal bacterial exposure promotes iNOS expression, which further enhances IgA (a major class of immunoglobulin) secretion by intestinal B cells, which is beneficial for promoting the intestinal barrier function. Conversely, iNOS acts as a microbicidal mediator, reducing microbial growth, and indirect antimicrobial effects are suggested to be caused by local arginine depletion after induction of iNOS or NO-dependent induction of IFN-γ (Bogdan, 2001). In another report, increased iNOS stimulated by quinolinic and picolinic acids together with 3-hydroxykynurenine and 3-hydroxyanthranilic acids enhanced lipid peroxidation and activated an arachidonic acid cascade, resulting in the production of inflammatory factors such as prostaglandins and leukotrienes (Oxenkrug, 2010). Overall, the iNOS pathway acts as a mediator between the gut microbiota and host immune system, and it is also related to Trp metabolism. Nonetheless it remains unclear how the iNOS pathway and Trp metabolism simultaneously participate in this mutual interaction, and further elucidation is required.

The microbiota influences host IDO and Trp metabolism through KP, though there are conflicting results for the same or different experiments. For example, IDO and KP metabolism decrease due to the deficiency in the microbiota in GF animals, whereas GF animals colonized with a normal microbiota or *T. gondii* have increased IDO and KP metabolism. However, colonization of probiotics (*B. infantis* and *L. johnsonii*) in conventional rodents reduced IDO activity and KP metabolism. The reason for these differences may be variation in the original gut microbial background in the transplanted animals, with one being GF and others conventional. Another reason may be the different types of colonized microbes. In relation to both experimentation and therapy via IDO activity regulation, these factors should be further investigated.

#### Kynurenines

Kynurenines possess antimicrobial activities, which can directly impact proliferation of the gut microbiota (Niño-Castro et al., 2014). The influence of the gut microbiota on host Trp metabolism in KP is associated with the immune system. GF animals that lack a microbiota have an immature immune system, which is associated with reduced Trp metabolism in KP (Clarke et al., 2013). After intestinal microbiota colonization in GF animals, immune system function is reinstated, and aberrant KP metabolism is normalized (Clarke et al., 2013). GI Toll-like receptors (TLRs), which recognize microbial components in the GI tract, act as crucial junctions (Kawai and Akira, 2010; Wang et al., 2010). In the GF state, TLR expression is reduced, which is associated with increased KP metabolism that may be mediated by IFN-γ-dependent or -independent IDO1 induction (Clarke et al., 2012).

In KP, kynurenine, KA, CA, and XA act as direct ligands of AhR, stimulating AhR and AhR-dependent gene expression in a concentration-dependent manner, and simultaneously modulate intestinal homeostasis. Additionally, AhR itself plays a role in regulating levels of IDO1 and TDO1 expression (Bessede et al., 2014). The absence of AhR causes an increase in endogenous KA levels in mice (García-Lara et al., 2015). AhR may be an important mediator in the complex crosstalk between the gut microbiota, KP and the immune response.

In addition, transmembrane G protein-coupled receptors (GPCRs) sense metabolic intermediates to activate signaling pathways and play roles in regulating GI homeostasis and intestinal immunity. GPCRs, including GPR43, GPR109A, and GPR120, exert anti-inflammatory effects (Tilg and Moschen, 2015). For instance, GPR43 deficiency induces severe inflammatory reactions in the mouse intestine (Maslowski et al., 2009). GPR35 is predominantly expressed in immune cells and in the GI tract, suggesting it may play an important role in immunological regulation. Trp metabolites, such as serotonin, melatonin, KA and niacin, are known GPCR ligands, and KA acts as a ligand for GPR35. Based on elevated KA levels, anti-inflammatory effects of KA during inflammation, and increased expression of GPR35 in immune cells, several studies have suggested that KA may have important functions in immunological regulation via GPR35 activation and subsequent signaling (Wang et al., 2006). Several bacteria can also catabolize Trp through KP (Genestet et al., 2014). Most strains of *P. aeruginosa* isolated from cystic fibrosis patients can produce a high level of kynurenine, which can promote bacterial survival and allow bacteria to circumvent the innate immune response by scavenging neutrophil reactive oxygen species (ROS) production (Genestet et al., 2014). Additionally, in *P. aeruginosa* KP, kynurenine acts as the main precursor of the *Pseudomonas* quinolone signal, which is another virulence factor of these bacteria (Genestet et al., 2014).

In summary, the interface for the gut microbiota, KP and immune response is tightly controlled and complex. Gut microbial composition plays an important role in regulating KP to subsequently influence host immunity, and variations in the composition of the gut microbiota can influence an individual's immunity and health through adjusted Trp metabolism in KP.

#### Serotonin

The serotonin pathway is one of the core signaling pathways in the gut (Gershon and Tack, 2007; Lesurtel et al., 2008). Serotonin plays a role in regulating the permeability of the intestine and mucosal inflammation. In the murine intestine, serotonin is associated with inflammation during chemically induced colitis. Suppressing the production of mucosal serotonin is beneficial for relieving inflammation (Margolis et al., 2013), and studies have found that GI-selective TPH (Trp hydroxylase) inhibitors may act as a cure for several GI diseases caused by serotonin pathway dysregulation (Shi et al., 2008). Changes in serotonin concentrations induced by the gut microbiota can regulate the host immune response and subsequently influence the coping strategy by which the host defends against pathogens or disease. Microbial SCFA metabolites can active GPCRs on intestinal epithelial cells and thus have a major role in regulating epithelial barrier integrity and intestinal immunity. At the same time, SCFAs promote *TPH1* transcription and colonic serotonin production from enterochromaffin cells and stimulate colonic transit, steps that are essential for serotonin homeostasis (Reigstad et al., 2015). Plasma serotonin levels were found to be decreased by 2.8-fold and levels of Trp increased in GF male Swiss Webster mice compared with conventional mice (Wikoff et al., 2009). At the same time, GF mice have decreased gut motility compared to normal animals, and decreased serotonin levels are one possible reason for this defect (Ridaura and Belkaid, 2015). In another experiment involving GF Swiss Webster mice, the concentration of hippocampal serotonin was significantly increased in GF and colonized GF animals compared with conventional mice (Clarke et al., 2013). The possible factors responsible for differences in serotonin levels in GF mice is worth further study. When discussing the effect of microbial colonization on host 5-HT levels, it is also important to consider the influence of altered IDO1 activity and KP metabolism induced by colonization, as lower IDO activity may result in decreased kynurenine and increased 5-HT concentrations.

Colonization of GF animals with the gut microbiota from humans or other mice can significantly increase gut motility, and this can be partially blocked by a pharmacologic antagonist of serotonin receptors (Kashyap et al., 2013). Indeed, serotonin can promote immunity and inflammation in various models of mucosal infections. Combining its effects on intestinal physiology and the gut microbiota, serotonin has been suggested to directly and indirectly influence GI motility and the immune system, which in turn shapes the composition and localization of the microbiota.

#### Melatonin

Melatonin acts as a powerful anti-inflammatory molecule, and the role of melatonin in the gut, especially its role in gut permeability, has recently been explored (Anderson and Maes, 2015). Melatonin release in the gut is 400-fold higher than that in the pineal gland (Bubenik et al., 1977), with a peak after food intake. Melatonin has positive impacts on gut disorders, including inflammatory bowel disease (IBD) (Eliasson, 2014) and GI cancer (Glenister et al., 2013).

The impact of melatonin on the gut microbiota has been examined. Specific gut bacteria determine the availability of Trp to the host and then regulate serotonin and subsequent melatonin synthesis (Wikoff et al., 2009). Moreover, melatonin can alleviate the increase in gut permeability and immune activation induced by *Escherichia coli* (Sun et al., 2013).

Inflammasomes, such as NOD-like receptor 3 (NLRP3) and pyrin domain-containing 6 (NLRP6), are known to be important effectors of gut permeability and interactions with gut bacteria. These inflammasomes are important for maintaining gut homeostasis (Zambetti and Mortellaro, 2014), including regulating gut permeability, and are crucial for IL-1b and IL-18 release. Activation of the alpha 7 nicotinic acetylcholine receptor (α7nAChr), an inflammasome activator in lipopolysaccharide (LPS) models (Kim et al., 2014), or melatonin (Galley et al., 2014) can reduce the incidence of sepsis by decreasing NLRP3 activation. Melatonin is a significant positive regulator of α7nAChr (Markus et al., 2010), suggesting that several regulatory effects of melatonin may be mediated by α7nAChr induction in the gut. For example, the protective effects of melatonin against induced gut permeability is mediated, at least in part, by α7nAChr (Sommansson et al., 2013). Several potential mediators that connect melatonin, gut bacteria, and gut immunity have been discovered, but the specific mechanisms of these connections remain to be determined.

### Bacterial Trp metabolites

Bacterial Trp metabolites, such as indole and indolic acid derivatives, are potent bioactive metabolites that affect intestinal barrier integrity and immune cells in mice by activating the pregnane X receptor (PXR) or aryl hydrocarbon receptor (AhR) (Zelante et al., 2013; Venkatesh et al., 2014; Lamas et al., 2016). The predominant Trp microbial metabolites in the intestine are indole, indole propionic acid, indole acetic acid, skatole, and tryptamine (Figure 1) (Yokoyama and Carlson, 1979). The gut microbiota can directly influence the type and level of Trp microbiota-derived metabolites, which can target host AhR, and subsequently modulate the mucosal immune response (Levy et al., 2016) or regulate mucosal integrity through PXR. Moreover, via AhR, bacterial Trp metabolites can modulate the production of IL-22, which plays a key role in intestinal homeostasis.

In addition, AhR- and PXR-regulated pathways are relevant for expression of the mucin 2 (*Muc2*) gene in the intestine (Zelante et al., 2013; Venkatesh et al., 2014). PXR-deficient mice exhibit a leaky gut and reduced expression of *Muc2* in the small intestine (Venkatesh et al., 2014). Commensal bacteria can utilize mucins as an energy source, which is important for establishing a mucosal-associated niche for potential health-associated commensals (Wlodarska et al., 2017). The following sections will introduce the mechanisms underlying regulation by the main bacterial Trp metabolites of intestinal homeostasis and immune responses.

Host genes can also directly or indirectly modulate the production of microbial Trp metabolites and affect the composition and function of the gut microbiota. Lamas et al. found that the microbiota in caspase recruitment domain 9 (CARD9)-deficient mice lack Trp-catabolizing capacity and fail to metabolize Trp into metabolites that can act as AhR ligands (Lamas et al., 2016). Consistent with this, *Card9*^−/−^ mice have decreased levels of bacteria with Trp-catabolizing functions, such as *Lactobacillus reuteri* (Zelante et al., 2013), and are more susceptible to intestinal inflammation. However, intestinal inflammation and the lack of Trp-catabolizing capacity in *Card9*^−/−^ mice can be reversed by supplementation of *Lactobacillus* strains that are capable of metabolizing Trp (Lamas et al., 2016). Researchers have hypothesized that in *Card9*^−/−^ mice, the altered immune response has an effect on the composition of the microbiota. In turn, the altered microbiota influence the production of Trp microbiota-derived metabolites, affecting the host's intestinal homeostasis and leading to the loss of intestinal homeostasis and intestinal inflammation.

#### Indole

Indole is a major bacterial Trp metabolite. The generation of indole is catabolized by tryptophanase, which can be induced by Trp or repressed by glucose in most bacteria (Figure 1C). Bacterial species including *E. coli, Proteus vulgaris, Paracolobactrum coliforme, Achromobacter liquefaciens*, and *Bacteroides* spp are capable of producing indole (Keszthelyi et al., 2009). Recently, indole has been recognized as a signaling molecule that can regulate bacterial motility, biofilm formation, antibiotic resistance, persister cell formation, and virulence (Li and Young, 2013) and that plays a role in affecting host cell invasion by other non-indole-producing species, such as *Salmonella enterica* and *P. aeruginosa* and even the yeast *C. albicans* (Li and Young, 2013). In the porcine gut with a low-non-starch polysaccharide diet, the maximum concentration of indole (~0.12 mM) was found in the distal part of the cecum, where the majority of gut bacteria had settled, whereas the amount of indole in the hind intestine was lower in animals fed a high-non-starch polysaccharide diet (Knarreborg et al., 2002). An explanation is that easily fermented carbohydrates such as non-starch polysaccharides, but not protein, are preferentially fermented by the intestinal microbiota, decreasing the production of indole from Trp degradation. Indole can also be detected in human feces, between 0.25 and 1.1 mM (consistent with the levels that are readily produced by *E. coli* when cultured in a rich medium), which suggests that intestinal epithelial cells are exposed to indole when the fermented substrate is available for Trp degradation in gut (Bansal et al., 2010).

As a specific bacterial signal, indole is abundant in the healthy mammalian gut and positively influences intestinal health. Indole has also been recognized as a beneficial signal in intestinal epithelial cells that can ameliorate intestinal inflammation in mammals (Bansal et al., 2010). Moreover, compared to other bacterial Trp metabolites, indole is the most effective molecule (Davis, 2014). Indole administration can attenuate damage of the GI tract induced by non-steroidal anti-inflammatory drugs (NSAIDs), modulating inflammation mediated by innate immune responses and alterations in the gut microbiota composition (Whitfield-Cargile et al., 2016). At millimolar concentrations, indole can weaken the invasion and colonization capabilities of enteric bacteria by reducing expression of *Salmonella* Pathogenicity Island-1 (SPI-1) genes, which facilitate bacterial invasion into host cells. After exposure to indole, the expression level of genes associated with strengthening the mucosal barrier and mucin production is increased, which is usually positively correlated with improvement in the resistance of human enterocyte HCT-8 cells. Indole exposure can also reduce TNF-α-mediated activation of NF-κB, expression of the proinflammatory chemokine IL-8, and the adherence of pathogenic *E. coli* to HCT-8 cells, though it does increase production of the anti-inflammatory cytokine IL-10. Variations in NF-κB activation and cellular resistance are highly specific to indole, as exposure to other indole-like molecules does not induce a similar response (Bansal et al., 2010).

Indole has been shown to promote the epithelial barrier functions of intestinal cells by fortifying epithelial tight junctions between cells through the pregnane X receptor (PXR) (Bansal et al., 2010; Shimada et al., 2013; Thaiss et al., 2016), which might contribute to resistance to inflammation. Indole can also enhance secretion of glucagon-like peptide-1 (GLP-1), an incretin with profound influences on host metabolism (Chimerel et al., 2014; Thaiss et al., 2016). As an important Trp microbiota-derived metabolite, indole can activate AhR signaling and subsequently promote local IL-22 production, which is important for intestinal homeostasis and further drives the secretion of antimicrobial peptides and protects against pathogenic infection (Levy et al., 2016). Considering these *in vitro* and *in vivo* results, it can be concluded that indole has beneficial effect on intestinal health. For normal cells, indole exposure can strengthen the mucosal barrier and mucin production by inducing expression of associated genes, thereby increasing resistance to pathogen invasion; for inflammatory cells, indole exposure can suppress activation of NF-κB and proinflammatory chemokine production and simultaneously increase anti-inflammatory cytokine production, thus ameliorating inflammation and damage.

As Trp can induce the enzyme tryptophanase, exogenous Trp levels influence the production of indole in *E. coli* in the gut (Li and Young, 2013). High-protein diets have also been reported to induce bacterial tryptophanase activity, which can result in overproduction of indole in the colon. Because indole affects bacterial physiology in a concentration-dependent manner, the level of indole that can be produced by microbiota is noteworthy. For example, 0.5 mM indole affects the motility of bacteria, the formation of biofilm, and the secretion of several virulence factors. Higher indole concentrations (1–2 mM) influence expression of multidrug exporters and several virulence factors; even higher indole concentrations (3–5 mM) can inhibit cell division and affect plasmid stability (Li and Young, 2013). Regarding the relationship among bacterial physiology, intestinal homeostasis and intestinal inflammation, we suggest that several nutritional factors, such as a high-protein or high-Trp diet, may impact intestinal homeostasis and intestinal inflammation via indole as an intermediary. This may constitute an effective approach to increasing the production of indole to modulate intestinal immunity through nutritional regulation.

#### Indolic acid derivatives

In the gut, a portion of Trp can be catabolized to indolic acid derivatives by bacteria, including indole-3-acetic acid (IAA), indole-3-aldehyde (IAld), indole acryloyl glycine (IAcrGly), indole lactic acid, and indole acrylic acid (IAcrA) (Figure 1) (Keszthelyi et al., 2009). Several intestinal bacteria, such as *Bacteroides, Clostridia*, and *E. coli*, can catabolize Trp to tryptamine and indole pyruvic acid, which are then converted to indole-3-acetic acid, indole propionic acid, and indole lactic acid (Smith and Macfarlane, 1997). Indole-3-acetic acid can be further combined with glutamine to produce indolyl acetyl glutamine in the liver or oxidized to indole-3-aldehyde (IAld) through peroxidase-catalyzed aerobic oxidation (De Mello et al., 1980). Indolyl propionic acid can also be further converted to indolyl acrylic acid (IAcrA) and combined with glycine to yield indolyl acryloyl glycine (IAcrGly) in the liver or kidney (Figure 1) (Keszthelyi et al., 2009).

The effects of several indolic acid derivatives on the gut microbiota and intestinal homeostasis have been reported. Several *Peptostreptococcus* species are capable of producing IAcrA, which can suppress inflammation by promoting intestinal epithelial barrier function and mitigating inflammatory responses. After LPS stimulation, IAcrA enhances both IL-10 production and mucin gene expression. Mucins can be utilized as an energy source by commensal bacteria, and IL-10 acts as an anti-inflammatory cytokine (Hasnain et al., 2013). Therefore, IAcrA is suggested to have an important anti-inflammatory function in the intestine, and stimulating IAcrA production to promote anti-inflammatory responses has therapeutic benefits (Wlodarska et al., 2017). *Clostridium sporogenes* can metabolize Trp into indolyl propionic acid, which protects mice from DSS-induced colitis (Venkatesh et al., 2014). Indolyl propionic acid significantly enhances IL-10 production (an anti-inflammatory cytokine) after LPS stimulation and reduces TNF production (a proinflammatory cytokine). Indole-3-aldehyde (IAld), which is able to activate ILC3s to produce IL-22 via AhR, is abundantly produced by *L. reuteri* in the presence of Trp in the gut. Additionally, mutation of *L. reuteri* caused the loss of its capacity to produce IAld and induce IL-22 in the presence of Trp (Romani et al., 2014). Several indolic acid derivatives are toxic to the microbiota. For instance, certain indolic compounds are known to have bacteriostatic effects on gram-negative enterobacteria, especially the genera *Salmonella* and *Shigella*, Furthermore, indolyl acetic acid has been reported to inhibit the growth and survival of *Lactobacillus*, specifically *L. paracasei* (Nowak and Libudzisz, 2006). Indole-3-aldehyde (IAld), a metabolite produced from Trp by commensal lactobacilli, can act as a ligand of AhR and subsequently activate AhR-dependent *IL-22* transcription (Zelante et al., 2013). IL-22 mediates pivotal innate antifungal resistance in mice (De Luca et al., 2010) and humans (Puel et al., 2010) and provides colonization resistance against the fungus *C. albicans* and mucosal protection from inflammation. Several authors have suggested that indolyl acryloyl glycine (IAcrGly) can increase intestinal epithelial permeability, and it has been hypothesized that increased intestinal permeability is caused by membrane damage induced by the precursor of IAcrGly: indolyl acrylic acid. Such membrane damage results in increased permeability and permeation of compounds that can disrupt normal intestinal homeostasis (Keszthelyi et al., 2009).

What can influence the production of these derivatives? Increased and prolonged excretion of urinary indolic acid derivatives has been detected in a number of diseases, such as Hartnup disorder, celiac disease and other malabsorption syndromes (Haverback et al., 1960). However, the effects of increased indolic acid derivatives on the immune system, especially on intestinal immunity, have not been evaluated in these diseases, and doing so may be useful for interpreting pathology and improving available therapies. Additionally, excessive Trp overload in the colon and concomitant gut microbiota alteration has been hypothesized to increase the production of Trp microbiota-derived metabolites, though this is merely a hypothesis. The types of metabolites that can be accelerated and whether other factors can influence production need to be validated.

#### 3-methylindole (skatole)

Skatole is another intestinal Trp microbiota-derived metabolite (Jensen et al., 1995), the precursor of which is indole-3-acetic acid (IAA) (Figure 1B) (Yokoyama and Carlson, 1979). Intestinal microbiota convert Trp to indole and IAA, and skatole is then synthesized from IAA via decarboxylation (Figure 1B). However, the level of skatole produced is usually low. Compared to other Trp bacterial-derived metabolites, skatole concentrations in the intestine are highly variable, which may be due to the two-step production process mediated by at least two different bacterial species; the concentration of the intermediate IAA may be another rate-limiting factor (Yokoyama and Carlson, 1979). *Lactobacillus, Clostridium*, and *Bacteroides* can convert IAA to skatole (Cook et al., 2007; Whitehead et al., 2008). The actual site of skatole production in the intestine is likely the small intestine and colon, and skatole can be efficiently absorbed by both. In pigs, the concentration of skatole in the colon is in excess of 30 μg/g (Yoshihara and Maruta, 1977). After oral supplementation with L-Trp, the concentration of skatole in bovine ruminal fluid is ~36 μg/ml. Skatole can influence the growth and reproduction of certain intestinal bacteria and has bacteriostatic effects on gram-negative enterobacteria. The genera *Salmonella* and *Shigella* are slightly more sensitive to the bacteriostatic effects of skatole than are *Escherichia* and *Aerobacter* species. In dilute solutions, skatole can also inhibit the growth and fermentation of *Lactobacillus acidophilus*. In this regard, skatole may determine the composition of intestinal microbes and the intestinal microbial ecosystem and protect the ecological niches of the bacteria that produce it (Yokoyama and Carlson, 1979). Regardless, the influence of skatole on host diseases through effects on intestinal microbes is not well-characterized.

The production of skatole is associated with both healthy and disease states. The fecal skatole concentration in humans varies considerably and may indicate different health statuses. Fecal skatole levels in healthy individuals are usually ~5 μg/g feces, whereas fecal skatole levels may be as high as 80 to 100 μg/g feces in persons who suffer from disturbed intestinal digestion (Yokoyama and Carlson, 1979). Researchers have suggested that after absorption but before detoxification, skatole may have a damaging effect on the activity and function of intestinal epithelial mucosa (Yokoyama and Carlson, 1979). In cattle, intraruminal and intravenous administration of skatole induces clinical features and lung lesions similar to Trp-induced disease. Skatole has been recognized as a primary cause for Trp-induced disease, which manifests as acute pulmonary edema and emphysema, generally resulting in death (Yokoyama and Carlson, 1979).

With an efficacy equivalent to that of indole, skatole exhibits modest dose-dependent activation to stimulate murine and human AhR. Within the GI tract, abundant generation of skatole may underlie the establishment of an axis to regulate intestinal physiology, which may involve microbiota-indole-AhR-mediated maintenance of intestinal homeostasis throughout a longer lifespan (Hubbard et al., 2015a). In another report, skatole has been described as an inhibitory factor for *CYP11A1*, leading to decreased formation of pregnenolone, which is the precursor of mineralocorticoids, glucocorticoids, and sex steroids (Mosa et al., 2016). In the gut, synthesis of endogenous steroid hormones, such as the anti-inflammatory glucocorticoid cortisol, is critical for the maintenance of intestinal homeostasis (Bouguen et al., 2015). Along with reduced *CYP11A1* expression (Coste et al., 2007) and decreased glucocorticoid production (Huang et al., 2014), disorders of intestinal steroidogenesis have been associated with IBD. Skatole has also been suggested to play a role in the disturbance of intestinal homeostasis and in the development of IBD via inhibition of *CYP11A1* expression and glucocorticoid production.

Nutrients are important factors that influence the concentrations of skatole in the intestine and other tissues. As indicated above, oral supplementation with Trp can induce high concentrations of skatole in bovine ruminal fluid. Sugar concentrations in the intestine are another factor. Decarboxylase is a key enzyme in the second step of skatole production, and under induction-repression regulation, sugar concentrations in the intestine have a significant impact on decarboxylase activity and subsequently influence skatole production (Yokoyama and Carlson, 1979). Antibiotic use is another important factor influencing skatole levels. The most sensitive regulator of the microbial population and conversions mediated by bacteria in the gut is the application of antibiotics (Engberg et al., 2000). For instance, pigs fed zinc bacitracin have reduced skatole concentrations in the blood and backfat compared with non-supplemented control pigs (Hansen et al., 2000). In addition, significant gender differences in both indole and skatole concentrations in the blood and backfat were observed (*P* ≤ 0·001) (Hansen et al., 2000). As skatole stimulates physiological responses in a dose-dependent manner, maintaining appropriate concentrations in the gut is beneficial for maintaining the intestinal microbial ecosystem, intestinal homeostasis and intestinal health.

#### Tryptamine

Tryptamine is produced by the decarboxylation of Trp, which is common in the plant kingdom but rare in bacteria (Figure 1A) (Williams et al., 2014). The common gut Firmicutes *C. sporogenes* and *Ruminococcus gnavus* are capable of decarboxylating Trp to tryptamine (Williams et al., 2014). Although Trp decarboxylation is rare in bacteria, the Human Microbiome Project demonstrated that at least 10% of the human population possesses at least one bacterium encoding a Trp decarboxylase among the intestinal microbiota (Williams et al., 2014). Therefore, the generation and physiological role of tryptamine in the gut is non-negligible.

Tryptamine, a β-arylamine, is a neurotransmitter with documented effects on intestinal motility that acts on the enteric nervous system to modulate intestinal homeostasis (Wlodarska et al., 2015). However, tryptamine can induce ion secretion by intestinal epithelial cells. In an experiment using an Using chamber, 3 mM tryptamine induced a marked change in short-circuit currents, indicating that it can influence colonic ion secretion, which subsequently regulates GI motility. It is hypothesized that tryptamine-mediated signaling might affect the transit of food particles and bacteria through the gut lumen (Williams et al., 2014). Tryptamine can also reduce the invasion and colonization capabilities of enteric pathogens, such as *Salmonella enterica* serovar Typhimurium (Davis, 2014). In addition, tryptamine exerts inhibitory activity against IDO1, which then influences immune surveillance. Upregulation of IDO1 is reported to be associated with the escape of malignant cells from immune surveillance (Muller and Scherle, 2006; Katz et al., 2008), and inhibition of IDO1 activity is regarded as an important target in interventions related to immune escape (Whiteside, 2006). Therefore, effects of tryptamine on IDO1 inhibition may contribute to a more effective tumor-reactive response by immune cells, which is considered part of a viable strategy for anticancer therapies (Tourino et al., 2013). Similar to other Trp metabolites, tryptamine acts as a ligand for AhR and activates AhR to regulate intestinal immunity (Islam et al., 2017). In turn, AhR plays a role in regulating tryptamine production when the intestinal balance is disturbed. For instance, in wild-type (WT) and *Ahr*-knockout (KO) mice, the concentrations of tryptamine in feces were similar in both Trp diet groups before DSS treatment. However, after DSS treatment, colonic tryptamine levels were markedly lower in *Ahr* KO mice compared to WT mice, which might be due to dysbacteriosis induction in the former (Islam et al., 2017). In addition, tryptamine is a ligand for trace amine-associated receptors (TAARs) and potentiates the inhibitory response of cells to serotonin (Zucchi et al., 2006). Tryptamine can also induce the release of serotonin (Takaki et al., 1985). Fluctuation in intestinal serotonin levels can modulate GI motility (Lundgren, 1998; Turvill et al., 2000) and is also involved in the pathology of IBD (Linden et al., 2003, 2005; Bischoff et al., 2009).

Alteration of tryptamine production is mediated by Trp-microbial metabolism. The concentration of tryptamine in feces increases ~3-fold in conventional vs. GF mice (Marcobal et al., 2013). Although only a small fraction of Trp is converted to tryptamine by the intestinal microbiota, levels of tryptamine can be drastically increased after Trp supplementation (Vikström Bergander et al., 2012). Indeed, a Trp-supplemented diet can increase tryptamine levels compared with a control diet. For instance, the concentrations of tryptamine in the colon and serum were found to be increased in Trp-supplemented diet mice compared to control diet mice (Islam et al., 2017). On the basis of these studies, Trp can be absorbed from the diet by microbes and then converted to tryptamine; the type and distribution of Trp metabolites are altered and influence the normal physiological function of the host intestine.

## Factors influencing Trp-microbiome-host immunity interactions in the gut

### Probiotics

**Figure 3:**
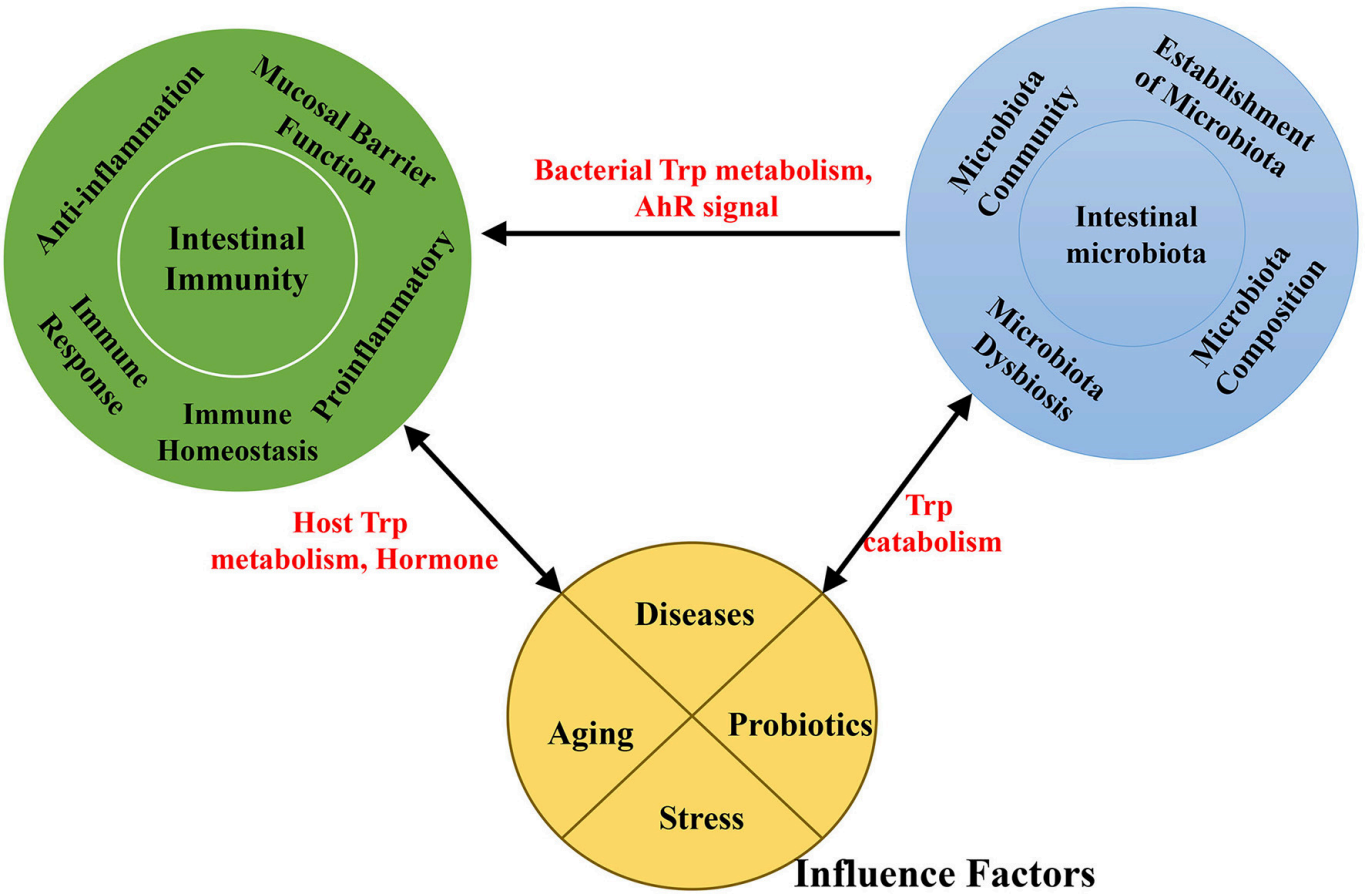
A schematic representation of the effects of interaction among the intestinal microbiota, intestinal immunity and factors associated with Trp absorption and metabolism.

Probiotics act as microbial food supplements that are beneficial to the host by improving the intestinal microbial balance. Studies indicate that probiotic supplements can modify the gut microflora and provide a practical means of enhancing gut and systemic immune function (Figure 3) (Nagata et al., 2016).

A particular focus has been the function of probiotics in Trp metabolism meditated by gut microbiota (Figure 3). Probiotics can selectively influence Trp metabolism and Trp concentrations. In bifidobacteria-treated rats, concentrations of plasma Trp (12.34 ± 0.87 vs. 8.03 ± 0.81 μg/ml, *p* < 0.005) and kynurenic acid (19.5 ± 3.7 vs. 7.9 ± 1.4 ng/ml, *p* < 0.05) were markedly increased compared with controls (Desbonnet et al., 2008). At the same time, bifidobacteria treatment significantly attenuated levels of proinflammatory cytokines, including IFN-γ, TNF-α, and IL-6, after mitogen stimulation in rats compared with untreated controls. These findings provide evidence that bifidobacteria treatment attenuates proinflammatory immune responses by elevating plasma Trp and kynurenic acid levels (Desbonnet et al., 2008). In an immunologically permissive environment, using the probiotic *L. reuteri* can effectively enhance the production of IAld through the Trp catabolism pathway when the substrate Trp can be accessed by the probiotic bacteria (Zelante et al., 2013; Marsland, 2016). As mentioned above, IAld, an AhR ligand, activates AhR in gut-resident T-cells and in ILCs, enhancing IL-22 production and protecting against inflammation in the colon. In addition, treatment with the probiotic *Lactobacillus fermentum* VRI-003 induced a certain increase in the IFN-γ response, a potent stimulus for IDO1 (Cox et al., 2010).

In what way might probiotic treatment affect Trp availability and Trp metabolism? First, probiotics can influence the gut microbiome composition, which may directly affect downstream metabolism and immunoregulatory pathways; the composition of gut bacteria can thus regulate Trp metabolism and the availability of Trp and Trp metabolites (Jenkins et al., 2016), influencing the immune response in the gut. As a therapeutic strategy in susceptible hosts, probiotic treatment can alter the intestinal microbiota and increase the generation of AhR ligands via Trp metabolism, which can protect the host from intestinal inflammation (Etienne-Mesmin et al., 2017). For example, in susceptible *Card9*^−/−^ mice, administration of three commensal *Lactobacillus* strains with high Trp-metabolic activities restored intestinal IL-22 production and reversed susceptibility to colitis (Lamas et al., 2016). However, only a small portion of probiotics have been examined for their ability to regulate intestinal immune function by affecting Trp availability and metabolism; moreover, the key genes involved in the probiotics have not been verified. Thus, more types of probiotics should be tested. New probiotics carrying specific Trp-metabolism genes through genetic engineering may be an important direction. However, more studies will be necessary to address these possibilities.

### Stress

The interaction between gut bacteria and regulation of the stress response is bidirectional (Figure 3). Physical and psychological stress can alter the composition of the GI microbiota in rodents and primates (Figure 3) (Bailey, 2014). In addition, manipulation of the intestinal microbiota has been shown to induce behavioral changes, including stress (Sudo et al., 2004), anxiety, and depression (Wilson et al., 2017). Stress responses of GF mice can be partially ameliorated by bacterial colonization with fecal matter from specific pathogen-free (SPF) mice to GF at early stages (Sudo et al., 2004).

Trp metabolism is closely related to stress-related challenges in animals. Stress, stress hormones and related neuropeptides have impacts on cerebral uptake of Trp, on serotonin synthesis, release and metabolism, and on other Trp-metabolism pathways. In detail, stress-induced increases in serotonin release in the brain is related to enhanced *Tph1* expression. Stress-related glucocorticoid hormones can induce TDO expression and activity at both the mRNA and protein levels (Ruddick et al., 2006), and immune stress induces IFN-γ release, which activates IDO expression and enhances NAD^+^ synthesis in macrophages through quinolinic acid as a substrate (Grant et al., 1999; Ruddick et al., 2006).

In addition, altered IDO1 activity and Trp metabolism are involved in many stress-related disorders, and stress-induced cortisol increases IDO1 activity and gut permeability. Individuals vulnerable to high levels of stress may benefit from Trp supplementation in the form of Trp-rich albumin or whey protein hydrolysates (Clarke et al., 2013). Nonetheless, little attention has been given to the possible connections among altered and affected Trp metabolites after stress inducement. Therefore, a rational strategy to alleviate stress-related disorders by adjusting and controlling Trp uptake and metabolism may be misguided.

Exhibiting a property of exaggerated stress reactivity, GF animals represent an effective model for studies to reveal the impact of the GI microbiota on Trp metabolism in response to stress (Figure 3). In GF animals, the kynurenine:Trp ratio is significantly decreased compared to conventionally colonized controls. This is however sex specific: the concentration of plasma Trp is increased in male GF animals (but not in females), and the concentrations of hippocampal 5-HT and 5-hydroxyindoleacetic acid are significantly elevated (again, not in females). After gut microbiota colonization in GF animals, the plasma Trp concentration was reduced and the kynurenine:Trp ratio increased compared with GF animals; plasma serotonin levels were also increased 2.8-fold (Wikoff et al., 2009). In addition, colonization by intestinal microbiota normalized stress in GF animals, displaying a more normal stress response. These results underline the ability of the microbiota to control Trp metabolism and the serotonergic system, which is particularly relevant to stress response and anxiety. Regardless, there are limited data pertaining to the relationship between plasma Trp concentrations and the kynurenine:Trp ratio, between plasma Trp and 5-HT concentrations, and between the same gut microbiota condition and the sex-specific serotonergic system.

The microbiota is suggested to play some role in regulating 5-HT synthesis, which is potentially mediated by IDO expression and functions in the stress response (Forsythe et al., 2010). Stress in the gut can alter the barrier function, enhance gut permeability and increase pro-inflammatory cytokines such as IL-1 and IL-6, which in turn alter IDO activity and Trp availability. Moreover, pro-inflammatory cytokines together with 5-HT influence the release of corticotropin-releasing hormone and vasopressin, which disorder the pressure response. Probiotic administration is shown to influence the stress response, and certain probiotic bacteria can alter the gut barrier function and 5-HT synthesis. Thus, probiotic administration is deemed a potential therapeutic for stress-related GI disorders such as irritable bowel syndrome (IBS). A more detailed understanding of the effects of stress on the induction of microbiome-gut-Trp alterations is needed, which may contribute to knowledge on the pathogenesis of several intestinal-related diseases.

### Aging

Aging is related to changes in the gut microbiota, which is frequently linked to physiological changes in the GI tract, together with a decline in immune system function that may contribute to increased risk for infection, malnutrition, and other functional deficiencies (Salazar et al., 2016) (Figure 3). The gut microbiota of elderly individuals is usually characterized by reduced bacterial diversity, altered dominant species, reduced beneficial microorganisms, and increased facultative anaerobic bacteria (Salazar et al., 2014), all of which indicate potential detrimental effects of microbial changes associated with aging. Changes in microbiota composition are connected to immuno-senescence and inflammation in older individuals (Franceschi et al., 2000; Biagi et al., 2013; Cheng et al., 2013).

Trp metabolism is affected by aging (Rampelli et al., 2013). Trp plays crucial roles in the induction of immune tolerance and the maintenance of gut microbiota (Figure 3). Analysis of Kyoto Encyclopedia of Genes and Genomes (KEGG) orthologous genes of intestinal core microbiomes in the elderly and young has shown that age-related genes with increased abundance are involved in the Trp metabolism pathway (ko00380) (Rampelli et al., 2013), which is consistent with the age-related reduction in Trp concentrations found in the serum of centenarians (Collino et al., 2013). Studies have suggested that a potential increase in Trp consumption by the gut microbiota may affect Trp bioavailability to the host (Rampelli et al., 2013). One recent study reported a relationship between reduced serum Trp levels and increased immune activation. For example, patients with inflammatory diseases show a significant depletion in serum Trp levels compared with healthy individuals (Gupta et al., 2012). It has also been speculated that a microbiota-dependent reduction in Trp enhances inflammation in centenarians (Rampelli et al., 2013).

In rats and mice, dietary Trp restriction is associated with a delayed aging process and prolonged lifespans (Van Beek et al., 2016). The accelerated aging *Ercc1*^—/*Δ7*^ mouse, which exhibits characteristics of normal murine aging, has been used as a model to research the relationship among aging, the gut microbiota, and Trp metabolism (Gurkar and Niedernhofer, 2015). Compared with wild-type mice, *Ercc1*^—/*Δ7*^ mice possess decreased microbial diversity, consistent with the reduced microbial diversity observed in aging humans (Biagi et al., 2012). Dietary Trp restriction can increase gut microbial diversity and cause the gut microbiota composition of older *Ercc1*^—/*Δ7*^ mice to be more similar to that of young wild-type mice, which might provide a valuable nutritional intervention strategy to improve age-related decreases in gut microbial diversity. At the same time, dietary Trp restriction can arrest B cell development in the bone marrow of 16-wk-old wild-type and *Ercc1*^—/*Δ7*^ mice. After dietary Trp restriction, decreased abundances of *Alistipes* and *Akkermansia* spp., which are both known to express tryptophanase, were positively correlated with decreased numbers of B cell precursors (Van Beek et al., 2016). In conclusion, dietary Trp restriction is a powerful intervention to modulate immunity, gut microbiota and aging. That is, aging is an important factor influencing host immunity, gut microbiota, and Trp metabolism. However, a beneficial interplay between dietary Trp, B cell development, and gut microbiota during aging can only be concluded, and there is no direct evidence for whether increased microbial diversity induces arrested B cell development or whether decreased B cell precursors cause changes in gut microbiota composition. In the gut microbiota, specific types of bacteria, *Alistipes* and *Akkermansia* spp., positively correlate with B cell precursors, though the key metabolites and the mode of action mediating this connection is unclear. Effects may include alleviating the harmful effects of aging or even slowing the aging process.

### Diseases

**Figure 4:**
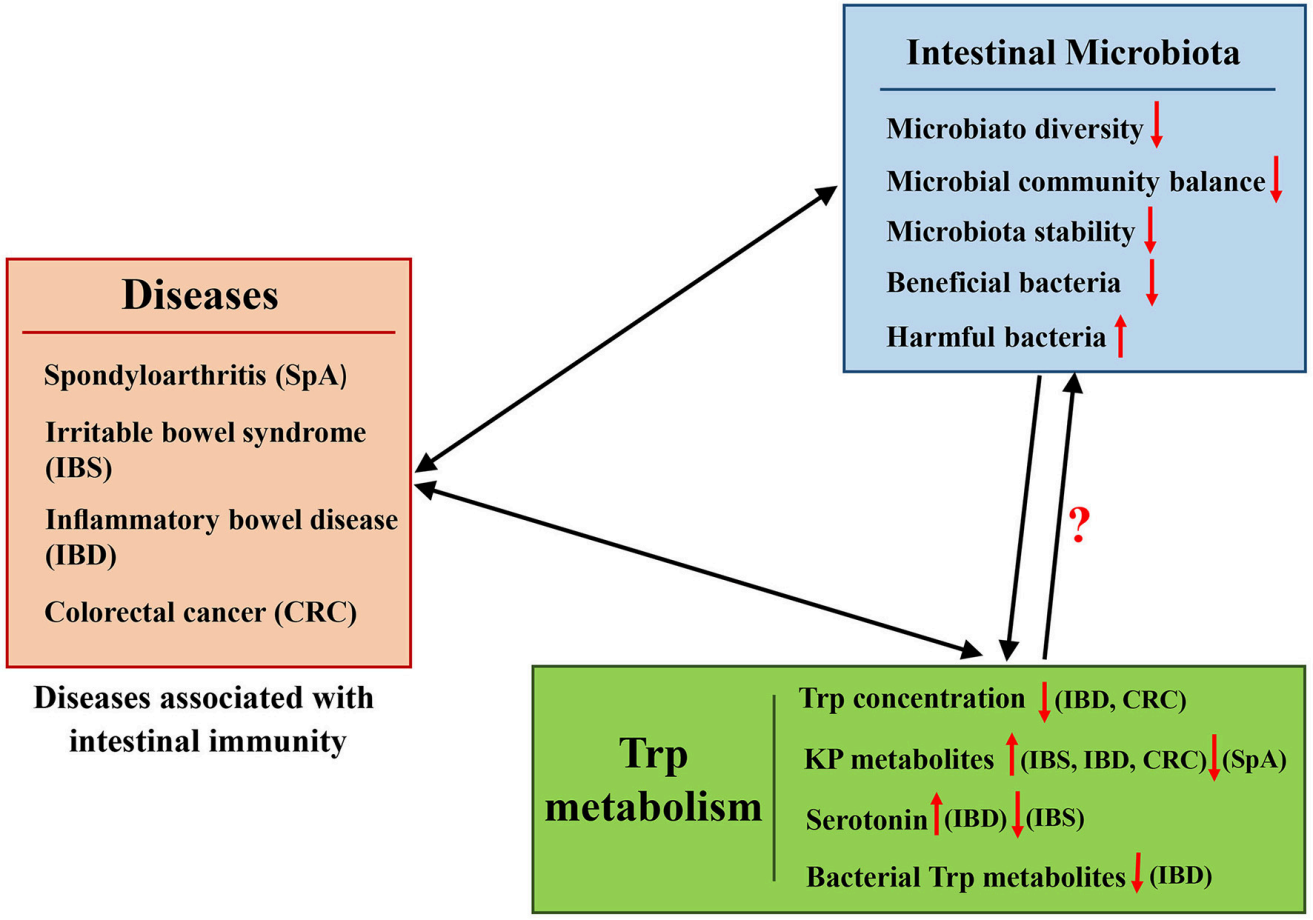
Representation of the effects of interaction between the intestinal microbiota and Trp metabolism in diseases associated with intestinal immunity.

The important role of the gut microbiota in host physiology and pathology has been extensively studied. A series of immune diseases, such as pediatric spondyloarthritis (SpA), IBS, IBD, and colon and GI cancer are associated with the gut microbiota (Uronis et al., 2009) (Figure 4). Trp metabolites also play important roles in regulating these immune diseases (Maes et al., 2007). As described above, the composition of the gut microbiota can influence Trp metabolite levels, and they both profoundly affect the immune status of the host, especially in the intestine.

#### Spondyloarthritis (SpA)

Spondyloarthritis (SpA) occurs in approximately one percent of the population in the United States (Lawrence et al., 2008). Half of SpA patients can have intestinal inflammation but not overt gastrointestinal symptoms (also called subclinical inflammation) (Vereecke and Elewaut, 2017). Based on the close relationship observed between SpA and intestinal inflammation, the microbiota has been suggested to be a potential factor in influencing immune responsiveness in SpA patients (Fantini, 2009; Scher et al., 2015). In pediatric or adult SpA patient feces, taxonomic differences in bacteria have been identified compared to healthy individuals (Costello et al., 2014; Stoll et al., 2014).

The influence of the gut microbiota in SpA has been studied (Lawrence et al., 2008; Costello et al., 2014; Stoll et al., 2014). With the decreased gut microbial diversity in pediatric SpA, relatively reduced Trp metabolism and lower levels of Trp metabolites in patients have been reported. The decrease in Trp metabolites in SpA patients is consistent with a similar finding in the synovial fluid of rheumatoid arthritis patients (Kang et al., 2015). Changes in fecal metabolomics and gut microbiota differences in Trp metabolism in pediatric SpA have been ascribed to alteration in the gut microbiota (Stoll et al., 2016). In the Trp metabolism pathway, 21 unique Trp metabolites were found to be reduced in the feces of SpA patients, as detected by charged ions, including the following: three bacterial Trp metabolites (indole-3-acetate, indole-3-acetaldehyde, and methyl indole-3-acetate); eight metabolites of kynurenine (such as 3-hydroxy-L-kynurenine, anthranilate, glutaryl-CoA, kynurenate); and six metabolites of 5-hydroxy-Trp. In addition, 16S sequence data of the fecal microbiome verify the relative decrease in Trp metabolism in SpA patients compared with controls (Stoll et al., 2016). Based on the above-described reports, the gut microbiota in enthesitis-related arthritis (one type of SpA) patients potentially acts as a proinflammatory factor due to altered Trp catabolism. As reported above, quite a lot of these Trp metabolites are associated with intestinal immune and intestinal inflammation, but it remains to be investigated which ones play dominant roles in causing subclinical inflammation in SpA. At the same time, which genes and gut microbes that target the specific lesser Trp metabolites should be further analyzed.

#### Irritable bowel syndrome (IBS)

IBS is one of the most commonly diagnosed chronic functional GI disorders, with a prevalence of ~15% in western populations (Soares, 2014). The pathophysiology of IBS is related to intestinal epithelial cells, enteroendocrine cells, neuronal cells, and immune cells. Alterations in gut microbiota diversity and composition have been implicated in IBS. Focusing on the symptomatology of IBS, changes in the intestinal microflora balance are an important factor (Nakai et al., 2003; Spiller, 2008; Bhattarai et al., 2017). Increases in the *Firmicutes* to *Bacteroidetes* ratio (Clarke et al., 2009) and the abundance of *Streptococcus* and *Ruminococcus* species are observed in IBS patients (Hong and Rhee, 2014), whereas *Lactobacillus* and *Bifidobacterium* populations (Balsari et al., 1982) are decreased.

In addition, IBS is associated with increased Trp metabolism via KP (Jenkins et al., 2016). The kynurenine:Trp ratio is positively related to symptom severity in IBS (Fitzgerald et al., 2008), and IFN-γ activation and subsequent IDO1 oxidation of Trp may be a pathogenic mechanism of IBS (Fitzgerald et al., 2008). In addition, dysfunction of the serotonergic system is associated with the pathophysiology of IBS. Serotonergic modulation through acute Trp increase treatment in IBS patients induced more severe GI symptoms compared with acute Trp depletion treatment (Kilkens et al., 2004; Shufflebotham et al., 2006). In IBS, the severity of symptoms and alterations of both gut and brain serotonin concentrations are associated with changes in the microbiota balance (Jenkins et al., 2016). IBS patients have lower serotonin concentrations in the small intestine, but colonic serotonin production can be promoted by effects of bacterial products on enterochromaffin cells, such as SCFAs (Reigstad et al., 2015). At the genetic level, microbiota from humanized and conventional mice increased colonic Trp hydroxylase 1 (*Tph1*) (a rate-limiting enzyme for mucosal 5-HT synthesis) expression through the stimulatory activities of SCFAs. For instance, butyrate, a SCFA, can active *Tph1* expression in mice through the inducible zinc finger transcription factor ZBP-89 (Essien et al., 2013). In the intestinal tract, nearly all SCFAs are produced through bacterial fermentation. Concentrations of SCFAs in the cecum of GF mice were reported to be ~1 mmol/kg, whereas average concentrations in the conventional mouse cecum are ~125 mmol/kg. Moreover, the variety and concentrations of SCFAs in human feces vary widely among individuals, which may be attributable to the complex microbial community or dynamic diet composition (Høverstad and Midtvedt, 1986).

Microbial-origin stimuli and the gut serotonergic system may act as key factors influencing the symptomatology of IBS. To alleviate GI symptoms through serotonergic modulation in IBS patients, the *in vivo* effects of local concentrations and proportions of different colonic SCFAs on mucosal 5-HT homeostasis should first be investigated, as SCFAs affect *Tph1* expression in a concentration-dependent manner; how to balance mucosal 5-HT homeostasis by manipulating the complex microbial community is challenging for this strategy. In addition, more data about the coadjustment between SCFAs and gut serotonergic system are required.

#### Inflammatory bowel disease (IBD)

IBD, defined as a chronic, remitting, and relapsing inflammatory disorder of the gut, includes Crohn's disease (CD), and ulcerative colitis (UC) (Matsuoka and Kanai, 2015; Lamas et al., 2017). Multifaceted factors, including dysregulation of the mucosal immune system, an unbalanced gut microbial community, and disruption of the mucosal barrier, are related to the pathogenesis of IBD (Flint et al., 2012; Pillai, 2013; Kostic et al., 2014; Matsuoka and Kanai, 2015; Lamas et al., 2017; Liu et al., 2017). Of these factors, the gut microbial community is gaining more attention due to its influence on intestinal health.

The pathogenesis of IBD is associated with alterations in the composition of the intestinal microbiome, but whether these alterations are causal or a result of inflammation in IBD is still under dispute (Gkouskou et al., 2014). In several reports, the gut microbiota has been considered to trigger inflammation in IBD (Sartor, 2008), as short-term treatment with antibiotics can markedly alleviate intestinal inflammation (Sartor, 2004; Kostic et al., 2014). In addition, an altered microbiome and altered interactions between intestinal microbes and mucosal immunity have been suggested to cause increased intestinal permeability and inflammation in IBD (Sartor, 2004; Hold et al., 2014).

Reductions in bacterial diversity have been reported in IBD patients (Martin-Subero et al., 2015), and these are accompanied by increased intestinal permeability and enhanced intestinal bacteria infiltration, which can induce immune responses and ultimately systemic inflammation (Xavier and Podolsky, 2007; Martin-Subero et al., 2015). IBD patients exhibit reduced intestinal mucosal barriers and decreased mucin glycosylation, and the disproportionate increase in several mucolytic microbes in IBD is suggested to be partly due to bacterial adaptation to the altered mucin glycosylation (Png et al., 2010). Several specific bacteria have been associated with IBD. For example, a decrease in *Bacteroidetes* (Frank et al., 2007) and *Bacteroides* (Nemoto et al., 2012) levels are reported in IBD patients, as were increases in *Desulfovibrio* and *Bilophila* levels (Rowan et al., 2010; Jia et al., 2012). A positive correlation between the invasive potential of *Fusobacterium* and the severity of IBD in the host was also found (Strauss et al., 2011), suggesting that invasive strains of *Fusobacterium* may influence IBD pathology (Kostic et al., 2014). In contrast, several specific species of gut bacteria may have protective effects against IBD (Kostic et al., 2014). For instance, species of *Bifidobacterium, Lactobacillus*, and *Faecalibacterium* may protect the host from mucosal inflammation by downregulating inflammatory cytokines or stimulating expression of IL-10, an anti-inflammatory cytokine (Sokol et al., 2008; Llopis et al., 2009).

IBD is also associated with altered host and gut bacterial Trp metabolites. Plasma levels of kynurenine and KA are increased (Forrest et al., 2003) and concentrations of plasma Trp are decreased in IBD patients, and increased IDO1 activity has been reported in the peripheral blood and colon cells of IBD patients (Munn and Mellor, 2007; Martin-Subero et al., 2015). In IBD, increased pro-inflammatory cytokines, including IFN-γ, IL-1, and IL-6, have been suggested to induce Trp catabolic pathways to reduce plasma Trp levels and increase Trp catabolite levels (Martin-Subero et al., 2015). In CD patients and IBD animal models, increased 5-HT levels are found in inflamed mucosa, suggesting the important role of 5-HT in driving intestinal inflammation in IBD (Levin and Van Den Brink, 2014). Several specific gut bacterial Trp metabolites are also involved in the pathophysiology of IBD (Matsuoka and Kanai, 2015). In dogs with IBD, bacterial Trp metabolites (indole acetate and indole propionate) that are suggested to have anti-inflammatory functions in the intestine are significantly decreased (Honneffer et al., 2015). In IBD patients, levels of IAA (anti-inflammatory function in the intestine) are reduced in feces, suggesting that reduced bacterial Trp metabolism may contribute to the etiology of IBD (Lamas et al., 2016). Furthermore, in IBD patients, the abundance of bacteria that can cleave terminal fucose residues from intestinal mucins by utilizing α-L-fucosidases is significantly reduced, correlating with the reduced production of indole acrylic acid and indole-3-propionic acid from Trp (Wlodarska et al., 2017). Studies have also suggested a strategy of increasing indole acrylic acid production by restoring bacterial Trp metabolism in the intestine to promote anti-inflammatory responses in IBD patients, which may have beneficial therapeutic effects.

#### Colorectal cancer (CRC)

CRC is the third-most prevalent cancer that causes mortality. The effects of the gut microbiota on CRC progression have been examined. GF animals have lower tumor burdens compared to conventionally raised counterparts. Similarly, depletion of the gut microbiota through antibiotics has also been shown to reduce CRC occurrence (Grivennikov et al., 2012). Alterations in gut microbiota composition have been found in murine tumorigenicity studies. In CRC patients, the fecal microbiota demonstrate decreased temporal stability but increased diversity of *Clostridium leptum* and *Clostridium coccoides* subgroups compared with the control group (Grivennikov et al., 2012).

Trp metabolism via KP has proven to be a potent target for immunotherapy. KP is activated in multiple tumor types; IDO1 initiates KP and is expressed in the primary tumor and in infiltrating myeloid-derived cells in CRC (Uyttenhove et al., 2003; Ferdinande et al., 2012; Théate et al., 2015). IDO1 appears to be chronically activated in cancer patients (Huang et al., 2002; Weinlich et al., 2007). IDO1 is commonly overexpressed in CRC and often accompanied by reduced Trp levels and increased levels of KP metabolites (Liu et al., 2010; Walczak et al., 2011; Engin et al., 2015). As introduced in above, IDO1 plays an essential role in maintaining microbiota diversity, and IDO1 activation can reduce microbial proliferation. Hence, we suggest that overexpression of IDO1 in CRC may be the cause of alterations and decreased temporal stability of the gut microbiota. As depletion of the gut microbiota can reduce CRC occurrence, decreasing the microbial proliferation induced by IDO1 activation may constitute self-regulation as a defense against CRC. In fact, research has proven that blockage of IDO1 activity can directly enhance the immune capacity of tumor-bearing mice against the tumors (Uyttenhove et al., 2003; Muller et al., 2005). However, the influence of the blockage of IDO1 activity on the mouse gut microbiota was not discussed, and it remains unclear whether the mouse gut microbiota played a role in this IDO1-blockage treatment. Regardless, selective inhibition of IDO1 is proposed to upregulate cellular immunity, with therapeutic potential in cancer, including CRC, and the influence of IDO1 on the gut microbiota is a factor that must be taken into account.

IDO1 also functions as a mediator in host-microbe crosstalk, which has effects on the pathology of GI cancer. When mediating host-microbe interactions, expression of IDO1 can be induced by activation of several binding pattern recognition receptors (PRRs), especially TLR4 and TLR9 (Ciorba et al., 2010; Santhanam et al., 2016). For example, TLR4 can be activated by LPS, a primary bacterial toxin. The ability of LPS to activate PRRs and then IDO1, reducing the generation of LPS and other similar toxins by controlling gut microbes, may be important for reducing the risk of GI cancer. Furthermore, new antagonists for PRRs to reduce expression of IDO1 may be beneficial against tumors. Indoles, as Trp microbial metabolites, have particular relevance for Trp metabolism and CRC (Raman et al., 2013), and several indoles derived from dietary Trp can activate AhR, which has important roles in intestinal homeostasis and controlling GI cancer. The influence of the gut microbiota and Trp metabolism on CRC pathogenesis will guide the development of targets and new approaches for the prevention and treatment of CRC.

## Summary and perspective

Our knowledge of interactions between Trp metabolism, gut microbiota, and host immunity has been greatly expanded over the past several years. Growing evidence shows that Trp, its endogenous host metabolites (kynurenines, serotonin, and melatonin) and its microbiome-modulated metabolites (indole, indolic acid, skatole, and tryptamine) have profound effects on gut microbial composition, microbial functions, the host-microbiome interface, and interactions between the host immune system and intestinal microbiota. Correspondingly, the gut microbiota affect host Trp absorption and metabolism, and directly or indirectly regulate subsequent host physiological and immune responses. In previous studies about Trp nutrition in conventional animals, some different or conflicting results have been reported. More attention was given to variation trends of endogenous host metabolites, whereas the effects of bacterial Trp metabolites were often overlooked. Thus, further comprehensive analyses of targeted Trp metabolites and associated genes are essential for experimental preciseness, which may explain the contradictory results in the literature.

Effects on host immunity by factors such as aging, stress, probiotic intake, and several diseases are partially associated with Trp-microbiome-immunity interactions. The influence of the gut microbiota on Trp metabolism should be assessed when studying Trp nutritional supplementation or Trp therapeutic applications. Furthermore, feasibly designed therapies that target Trp absorption, Trp metabolites, the gut microbiota, or Trp-microbiome-immunity interactions are promising approaches for treating intestinal or extra-intestinal inflammation. Moreover, the complex and variable gut microbiota may allow for identifying simple universal rules about bacterial Trp metabolites, and personalized gut microbiota analysis may be an effective approach for instituting clinical treatment. Similarly, further studies are required to determine which gut microbial species, the effective dose ranges of Trp metabolites, and which metabolite-targeted pathways may impact local and systemic inflammatory processes in the GI tract.

## Author contributions

All the authors contributed extensively to the work presented in this manuscript. KX and JG mainly completed this review. KX, JG, and HL performed the literature search and wrote the manuscript. KX, MB, GL, CP, TL, and YY conceived the work and critically revised it. YY revised the manuscript.

### Conflict of interest statement

The authors declare that the research was conducted in the absence of any commercial or financial relationships that could be construed as a potential conflict of interest.
